# Real-World Effectiveness and Safety of Selective JAK Inhibitors in Ulcerative Colitis and Crohn’s Disease: A Retrospective, Multicentre Study

**DOI:** 10.3390/jcm13247804

**Published:** 2024-12-20

**Authors:** Bernadett Farkas, Talat Bessissow, Jimmy K. Limdi, Karishma Sethi-Arora, Anna Kagramanova, Oleg Knyazev, Cristina Bezzio, Alessandro Armuzzi, Milan Lukas, George Michalopoulos, Elena Chaskova, Edoardo Vincenzo Savarino, Fabiana Castiglione, Antonio Rispo, Eszter Schäfer, Simone Saibeni, Rafal Filip, Mohamed Attauabi, Fotios S. Fousekis, Péter Bacsur, Tamás Resál, Anita Bálint, Emese Ivány, Zoltán Szepes, Zsófia Bősze, Anna Fábián, Renáta Bor, Klaudia Farkas, Peter L. Lakatos, Tamás Molnár

**Affiliations:** 1 Center for Gastroenterology, University of Szeged, 6725 Szeged, Hungary; farkas.bernadett@med.u-szeged.hu (B.F.);; 2 Division of Gastroenterology, McGill University Health Centre, Montreal, QC H4A 3J1, Canada; 3 Northern Care Alliance NHS Foundation Trust, Manchester M6 8HD, UK; 4 Moscow Clinical Scientific Center Named after A. S. Loginov, Moscow 111123, Russia; 5 Research Institute of Health Organization and Medical Management, Moscow 115088, Russia; 6 State Scientific Centre of Coloproctology Named after A.N. Ryzhyh, Moscow 123423, Russia; 7 IBD Center, IRCCS Humanitas Research Hospital, 20089 Milan, Italy; 8 Department of Biomedical Sciences, Humanitas University, 20072 Milan, Italy; 9 Clinical and Research Centre for Inflammatory Bowel Diseases, 19000 Prague, Czech Republic; 10 General Hospital of Athens “G. Gennimatas”, 11527 Athens, Greece; 11 Department of Coloproctology, Irkutsk Regional Hospital, Irkutsk 664528, Russia; 12 Federal Scientific Center of Surgery and Traumatology, Irkutsk 664003, Russia; 13 Gastroenterology Unit, Azienda Ospedale Università of Padua, 35128 Padua, Italy; 14 Department of Surgery, Oncology and Gastroenterology, University of Padua, 35124 Padua, Italy; 15 Gastroenterology Department of Clinical Medicine and Surgery, University of Naples Federico II, 80131 Naples, Italy; 16 Department of Gastroenterology, Hungarian Defence Forces Military Hospital, 1062 Budapest, Hungary; 17 Gastroenterology Unit, Rho Hospital, ASST Rhodense, 20017 Milan, Italy; 18 Department of Gastroenterology with IBD Unit, Clinical Hospital 2, 35301 Rzeszów, Poland; 19 Department of Gastroenterology and Hepatology, Copenhagen University Hospital—Herlev and Gentofte, 2730 Herlev, Denmark; 20 Division of Gastroenterology, University Hospital of Ioannina, 45500 Ioannina, Greece; 21 HCEMM-USZ Translational Colorectal Research Group, 6725 Szeged, Hungary; 22 Department of Internal Medicine and Oncology, Semmelweis University, 1085 Budapest, Hungary

**Keywords:** ulcerative colitis, Crohn’s disease, upadacitinib, filgotinib, JAK-inhibitors

## Abstract

**Background/Objectives**: Data on the real-world effectiveness and safety of selective JAK inhibitors (JAKis) in ulcerative colitis (UC) and Crohn’s disease (CD) are limited. **Methods**: We conducted a multicentre, retrospective study to assess clinical, biochemical, and endoscopic outcomes of selective JAKis in bio-experienced UC and CD. **Results**: A total of 246 patients (mean age: 40.5 ± 14.5 years; 131 UC and 115 CD) were included with a median follow-up of 7.5 months. Among the CD patients receiving upadacitinib (*n* = 115), 76.2% achieved clinical remission (CR) at week 12. Furthermore, 59.5% of the upadacitinib-treated UC patients (*n* = 100) experienced CR at week 8. Corticosteroid-free CR (CSFCR) was achieved by 76.9% of the CD patients and 80.6% of the UC patients at week 24, while 50.0% and 36.1% experienced endoscopic remission. At week 52, 66.7% of the CD and 86.2% of the UC patients achieved CSFCR, whereas 54.5% and 52.9% had endoscopic remission. In UC, the effectiveness of upadacitinib was not compromised by prior tofacitinib failure, while the upadacitinib-treated CD patients with stricturing and penetrating disease were less likely to achieve CR by the end of the induction phase (*p* = 0.04). C-reactive protein (*p*[CD] < 0.0001; *p*[UC] < 0.0001) and faecal calprotectin (*p*[CD] < 0.0001; *p*[UC] = 0.02) decreased significantly in both patient groups as early as week 2. Among the filgotinib-treated UC patients (*n* = 31), 28.6% were in CR at week 12. At week 24 and 52, 59.1% and 60% achieved CSFCR, while 0.0% and 20.0% had endoscopic remission. Both C-reactive protein (*p* = 0.04) and faecal calprotectin (*p* = 0.04) decreased significantly by week 12. Hyperlipidaemia (9.7–9.8%) was the most common adverse event. **Conclusions**: Selective JAKis are rapidly effective and safe for treating refractory, moderate-to-severe CD and UC.

## 1. Introduction

Rapid advances in our understanding of the immune aetiopathogenesis of inflammatory bowel diseases (IBDs) in the last two decades have translated into a dramatic increase in our therapeutic armamentarium, with biological and small molecule therapies intercepting and abrogating the immune–inflammatory cascade. However, a significant proportion of patients do not respond favourably (primary non-response [PNR]), or eventually experience a loss of response (LOR) to these agents, often as a result of anti-drug antibodies or various pharmacokinetic and pharmacodynamic mechanisms [1]. Among novel small molecule agents, Janus kinase inhibitors (JAKis) offer rapid onset of action and no risk of immunogenicity [2,3,4,5].

Compared to previously approved biologics, JAKis can block multiple cytokine pathways, which could potentially improve their effectiveness [3]. Members of the JAK family (JAK1, JAK2, JAK3, and tyrosine kinase 2 [TYK2]) are involved in a variety of physiological functions, including the regulation of inflammatory response, haematopoiesis, and immunohomeostasis. The dysregulation of JAK/STAT signalling has been described in several systemic disorders, including IBD [6,7,8]. Previous studies on panJAKi tofacitinib (TOFA) have shown it to be an effective therapeutic option in moderate-to-severe UC patients, even after anti-TNF failure [9,10]. Presumably, the inhibition of JAK2 and JAK3 is associated with a higher risk of adverse events (AEs), leading to the introduction of selective JAK1 inhibitors, including upadacitinib (UPA) and filgotinib (FILGO) [7,11,12,13,14]. In contrast to UPA, which has been shown to be effective in both UC and CD in clinical studies, FILGO has not been approved for CD as the induction cohorts in the registrational trial (DIVERSITY) failed to meet the coprimary endpoints of endoscopic response and clinical remission at week 10 [15,16,17,18].

Although the data on the efficacy and safety of selective JAKis in clinical trials are promising [16,17,18,19,20], evidence from real-world studies in IBD patients remains limited [21,22,23,24,25].

To address this unmet need, we conducted a multicentre, retrospective, real-world study that aimed to evaluate the effectiveness and safety of selective JAKis in bio-experienced, moderate-to-severe UC and CD patients. Furthermore, we also aimed to assess whether the effectiveness of selective JAKis was affected by the prior failure of TOFA.

## 2. Materials and Methods

### 2.1. Study Design and Settings

Data were collected from 22 tertiary centres in Europe and Canada from July 2022 to June 2024. The required data were retrieved from local medical record systems and entered into a purpose-designed, unified database by all investigators. The follow-up time was estimated from the initiation of the selective JAKi to the last IBD-related medical visit in the study period.

### 2.2. Participants

Patients diagnosed with CD and UC (based on clinical, biochemical, endoscopic, and histological findings) ≥18 years of age were included [26,27]. Disease phenotype was defined according to the Montreal classification [28]. Clinical disease activity was evaluated using the partial Mayo score (pMayo) in UC and the Crohn’s Disease Activity Index (CDAI) in CD patients, while endoscopic disease activity was assessed using the Mayo endoscopic subscore (eMayo) and the Simple Endoscopic Score (SES-CD) [29,30,31]. The indication for selective JAKi treatment was moderately to severely active luminal CD or UC. All enrolled patients were treated with at least one biologic or small molecule agent before the initiation of the selective JAKi. Participants with acute severe UC (based on the Truelove–Witts criteria [32]) at the initiation of UPA or FILGO, and patients who were bio-naive or received UPA or FILGO for extraintestinal manifestations or for isolated perianal disease without any luminal activity, were excluded, as were those UC patients who underwent colectomy prior to selective JAKi therapy. Patients receiving selective JAKis in combination with other biologics or small molecules were also excluded. All participants were required to have at least 2 weeks of follow-up.

### 2.3. Data Collection

The following patient- and disease-related data were collected: date of birth, sex, smoking habits, date of IBD diagnosis, age at diagnosis, and disease extent and severity at diagnosis and during follow-up. The previous therapeutic regime was also documented. The type and duration of selective JAKi therapy and the reason for treatment discontinuation were recorded. Serum albumin, C-reactive protein (CRP), and faecal calprotectin (FC) levels were assessed at the time of treatment initiation (within one week) and during the follow-up, while the complete blood count, parenchymal liver enzymes, serum cholesterol, and triglyceride levels were examined at the start of treatment (within one week) and at week 12 post-initiation. The use of concomitant corticosteroids (CSs) was also noted, along with the need and date for dose escalation. Furthermore, the date and indication of surgical interventions were recorded. The type and severity of AEs that occurred during selective JAKi therapy were also documented. The need for IBD-related hospitalisations during the follow-up period was also assessed.

### 2.4. Outcomes

The primary outcome was to evaluate the short-term effectiveness of selective JAKi in bio-experienced, moderate-to-severe CD and UC patients through clinical outcomes (clinical response, clinical remission [CR]) at weeks 2, 8, and 12. The secondary outcome was to monitor the changes in FC and serum CRP levels during the course of treatment. The tertiary outcome was to assess the rate of corticosteroid-free clinical remission (CSFCR) and endoscopic remission at weeks 24 and 52. We also investigated various factors that may predict CR with selective JAKis at week 12 in CD and at week 8 in UC. In addition, we assessed treatment persistence with selective JAKis.

### 2.5. Definitions

Clinical response was defined as a decrease in pMayo from baseline of ≥30% or ≥3 points in UC patients, while in CD patients, it was defined as a reduction in CDAI scores of ≥70 points. CSFCR was defined as CR (CDAI < 150 in CD, and pMayo score ≤ 2 with a rectal bleeding subscore of 0 in UC) and not receiving local and systemic steroids ≥ 90 days before the assessment time point. Biochemical remission was strictly defined as a serum CRP level of ≤5 mg/L and an FC level of ≤150 µg/g. CRP and FC levels above these values were considered as biochemical activity. The definition of endoscopic response in UC patients was a decrease in the eMayo score of ≥ 1, and for endoscopic remission, an eMayo score of 0, while in CD patients, an endoscopic response was defined as a decrease in SES-CD > 50% from baseline, and endoscopic remission as an SES-CD of ≤4 points. Complete remission was reported in the presence of clinical, biochemical, and endoscopic remission. The Perianal Disease Activity Index (PDAI) was reported at each timepoint in CD patients with active perianal fistula.

### 2.6. Statistical Methods

All statistical analyses were carried out using the program SPSS (IBM Statistical Package for Social Sciences for Windows, version 29.0, IBM Corp., Armonk, NY, USA). Descriptive statistical analysis was presented as median with interquartile range (IQR) or mean with standard deviation (SD) for continuous variables. Categorical variables were summarised using frequency and percentage. Differences in continuous variables were assessed using the paired-sample *t*-test. The chi-square test was used to determine the associations between patient groups and categorical variables. Kaplan–Meier analyses were performed to assess the rates of treatment persistence. Demographic and clinical factors associated with the achievement of clinical remission at the end of the induction phase were analysed via univariable and multivariable logistic regression models. Variables from the univariable analysis with a *p* < 0.15 were fitted and entered into the multivariable analysis. Hosmer–Lemeshow goodness of fit test was created to determine the performance of the final model, and the value of Nagelkerke R2 was also reported. A *p*-value < 0.05 was considered significant. The therapeutic response was only assessed in patients who had required data available at the specific time point (“as observed analysis”). At each predetermined time point, we indicated how many of the patients with a sufficient follow-up had the necessary parameters to assess therapeutic response or had biochemical or endoscopic data available (“number of patients with available data/number of patients with sufficient follow-up”). Incomplete or missing data were not imputed. All patients who achieved remission were also counted as responders.

### 2.7. Ethical Approval

Ethical approval for this study was obtained from the National Institute of Pharmacy and Nutrition according to the Scientific Research Ethics Committee of the Hungarian Medical Research Council’s proposal (Registration number: NNGYK/GYSZ/19841-1/2024 and BM/13035-3/2024). This research was conducted according to The Code of Ethics of the World Medical Association (Declaration of Helsinki).

## 3. Results

### 3.1. Baseline Characteristics

**Figure 1:**
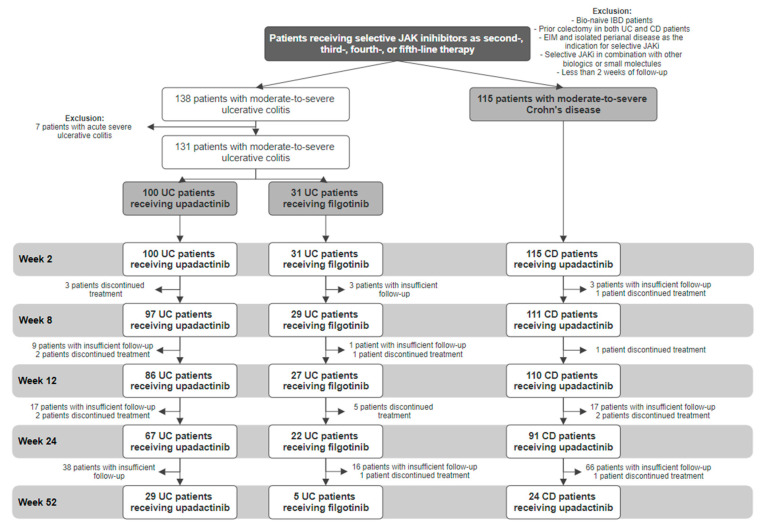
Flowchart for the enrolment and follow-up of participants.

A total of 246 patients (Figure 1; 131 UC and 115 CD; mean age: 35.3 ± 14.7 years; male/female ratio: 133/113) were included in this study.

The median follow-up time was 7.5 months (IQR: 5.1–11.6). Patient demographics and disease characteristics are shown in Table 1.

All patients received the dosage of selective JAKis recommended by the European Medicines Agency (UPA: 45 mg daily for induction, 15 mg/30 mg daily for maintenance; FILGO: 200 mg daily for induction, 100 mg/200 mg daily for maintenance).

### 3.2. Effectiveness of Selective JAK Inhibitors in Crohn’s Disease

**Figure 2:**
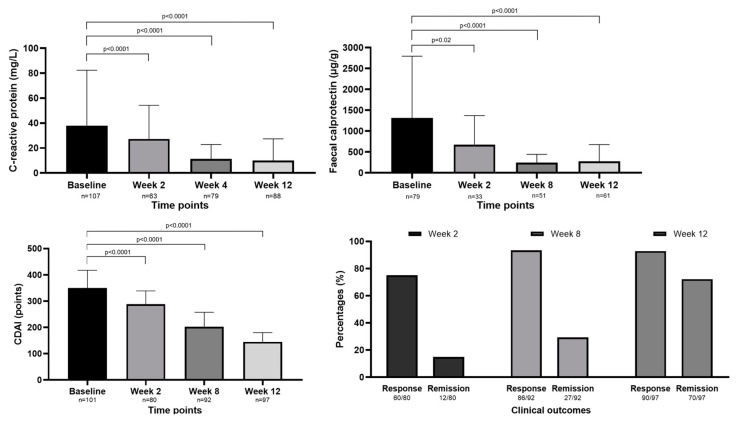
The changes in CDAI, C-reactive protein, and faecal calprotectin levels at the first 3 months of upadacitinib therapy in Crohn’s disease. The rate of clinical response and clinical remission at weeks 2, 8, and 12. The median CDAI decreased from 360 [418–267] at baseline to 288 [339–180] at week 2 (*p* < 0.0001), to 203 [258–133] at week 8 (*p* < 0.0001), and to 145 [180–100] at week 12 (*p* < 0.0001). The mean FC level decreased from 1379.0 ± 1267.0 µg/g at baseline to 1003.0 ± 716.6 µg/g at week 2 (*p* < 0.0001), to 525.7 ± 411.2 µg/g at week 8 (*p* < 0.0001), and to 388.1 ± 366.6 µg/g at week 12 (*p* < 0.0001). The mean CRP level decreased from 38.0 ± 44.4 mg/L at baseline, to 27.3 ± 27.1 mg/L at week 2 (*p* < 0.0001), to 11.3 ± 11.5 mg/L at week 8 (*p* < 0.0001), and to 10.0 ± 17.4 mg/L at week 12 (*p* < 0.0001).

A total of 115 CD patients received UPA. Of the 97/110 patients who had data on the therapeutic response at week 12, 92.8% (90/97) of the patients showed a clinical response, and 76.2% (70/97) achieved CR (Figure 2).

After the induction phase, 21.8% (24/110) of the patients received a maintenance dose of 15 mg, while 78.2% (86/110) were on a maintenance dose of 30 mg. The median CDAI (*p* < 0.0001) and mean FC (*p* < 0.0001) and CRP (*p* < 0.0001) levels decreased significantly from baseline during the first 3 months of UPA treatment (Figure 2). Data on both biomarkers were available for 33/80, 49/92, and 58/97 patients at weeks 2, 8, and 12, respectively. Biochemical remission was reported in 30.3% of (10/33) the patients at week 2, 22.5% (11/49) of the patients at week 8, and 29.3% (17/58) of the patients at week 12.

**Figure 3:**
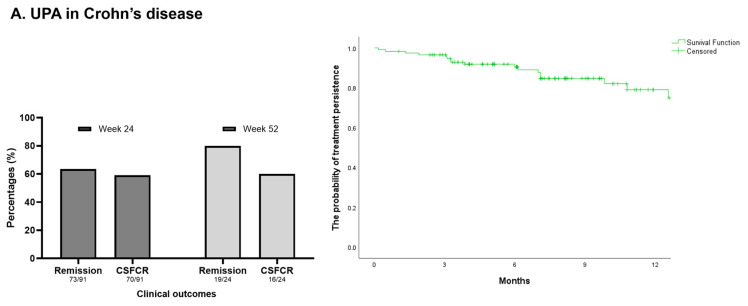

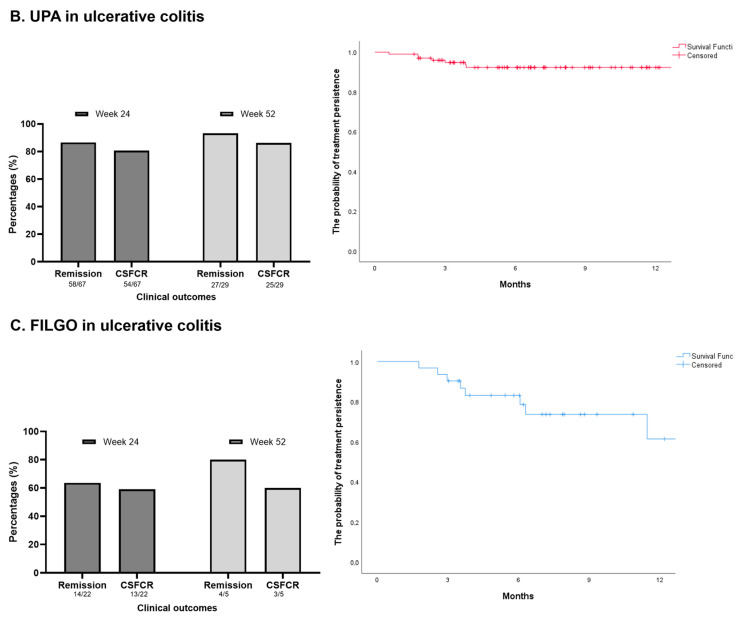
(**A**) The rate of clinical remission and corticosteroid-free clinical remission at weeks 24 and 52 in Crohn’s disease patients receiving upadacitinib. The probability of treatment persistence over 12 months post-initiation of upadacitinib. (**B**) The rate of clinical remission and corticosteroid-free clinical remission at weeks 24 and 52 in ulcerative colitis patients receiving upadacitinib. The probability of treatment persistence over 12 months post-initiation of upadacitinib. (**C**) The rate of clinical remission and corticosteroid-free clinical remission at weeks 24 and 52 in ulcerative colitis patients receiving filgotinib. The probability of treatment persistence over 12 months post-initiation of filgotinib.

At week 24, 91.2% (83/91) of the patients had a clinical response, and 80.2% (73/91) showed CR. CSFCR was documented in 76.9% (70/91) of the patients (Figure 3A).

Biochemical activity was observed in 53.4% (39/73) of the patients who had CR. At week 24, colonoscopy was performed in 46 patients, of which 58.7% (27/46) showed an endoscopic response and 28.3% (13/46) had endoscopic remission. Clinical, biochemical, and endoscopic data were available in 42/91 patients, of which 38.1% (16/42) had reached complete remission.

At week 52, 79.2% (19/24) of the patients experienced CR, while 66.7% (16/24) had CSFCR. (Figure 3A). In 52.6% (10/19) of the patients with CR, biochemical activity was detected. At week 52, endoscopy was performed in 11 patients. Overall, 63.6% (7/11) of the patients achieved an endoscopic response, while 54.5% (6/11) of the patients showed endoscopic remission. Clinical, biochemical, and endoscopic data were available in 11/24 patients, and 45.5% (5/11) had complete remission.

Clinical remission rates stratified by disease location are presented as Supplementary Material (Table S1). Among the 19 CD patients who had active perianal disease in addition to luminal activity at initiation of UPA therapy (median PDAI: 4 [11–2]), 13 (68.4%) patients achieved a PDAI score of 0 by week 24.

Dose escalation was reported in 41.7% (5/12) of the CD patients treated with 15 mg UPA, on average 5.9 (±2.4) months after treatment initiation. IBD-related hospitalisation was reported in 8.7% (10/115) of the patients during follow-up. Intestinal resection was required in 3.5% (4/115) of the patients. Seton drainage was required in two patients and fistula repair in one patient with perianal disease. The probability of treatment persistence with UPA was 90.5% and 79.0% at weeks 24 and 52, respectively (Figure 3A). UPA was discontinued in 16.5% (19/115) of the patients by the end of the follow-up period, mostly due to LOR (36.8%), PNR (26.3%), and AEs (26.3%).

### 3.3. Effectiveness of Selective JAK Inhibitors in Ulcerative Colitis

Overall, 76.3% (100/131) of the patients with UC were treated with UPA, while 23.7% (31/131) received FILGO therapy. Directly prior to initiating UPA, 45% (45/100) of the UC patients received TOFA, while all patients receiving FILGO were TOFA-naive.

#### 3.3.1. Upadacitinib

**Figure 4:**
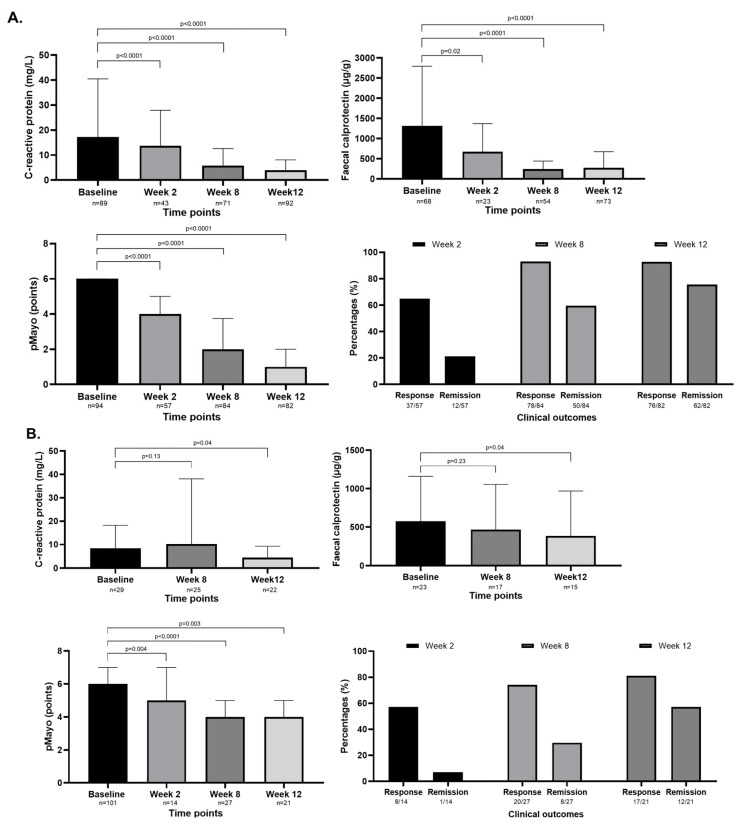
(**A**) The changes in pMayo and CRP and FC levels at the first 3 months of upadacitinib therapy in ulcerative colitis. The rate of clinical response and clinical remission at weeks 2, 8, and 12. The median pMayo decreased from 6 [6–5] at baseline to 4 [5–3] at week 2 (*p* < 0.0001), to 2 [4–1] at week 8 (*p* < 0.0001), and to 1 [2–0] at week 12 (*p* < 0.0001). The mean FC level decreased from 1319.0 ± 147.0 µg/g at baseline to 674.7 ± 697.1 µg/g at week 2 (*p* = 0.02), to 246.6 ± 197.8 µg/g at week 8 (*p* < 0.0001), and to 273.3 ± 403.0 µg/g at week 12 (*p* < 0.0001). The mean CRP level decreased from 17.2 ± 23.3 mg/L at baseline, to 13.7 ± 14.3 mg/L at week 2 (*p* < 0.0001), to 5.8 ± 6.8 mg/L at week 8 (*p* < 0.0001), and to 4.0 ± 4.2 mg/L at week 12 (*p* < 0.0001). (**B**) The changes in pMayo and CRP and FC levels at the first 3 months of FILGO therapy in UC. The rate of clinical response and clinical remission at weeks 2, 8, and 12. The median pMayo decreased from 6 [7–5] at baseline to 4 [7–5] at week 2 (*p* = 0.004), to 4 [5–2] at week 8 (*p* < 0.0001), and to 4 [5–2] at week 12 (*p* = 0.003). At week 2, no data on FC and CRP levels were available for any patients. The mean FC level decreased from 576.8 ± 584.6 µg/g at baseline to 466.5 ± 589.0 µg/g at week 8 (*p* = 0.23) and to 383.9 ± 586.1 µg/g at week 12 (*p* = 0.04). The mean CRP level increased from 8.4 ± 9.9 mg/L at baseline to 10.24 ± 27.9 mg/L at week 8 (*p* = 0.13), while it decreased to 4.5 ± 4.8 mg/L at week 8 (*p* = 0.04).

Data on therapeutic response were available for 84/97 patients at week 8, of which 92.9% (78/84) of the patients achieved clinical response and 59.5% (50/84) of the patients CR (Figure 4A).

The induction phase was extended to 16 weeks for 17.5% (17/97) of the patients. After the induction phase, 35.9% (32/89) of the patients received a maintenance dose of 15 mg, while 64.1% (57/89) were on a maintenance dose of 30 mg. The median pMayo (*p* < 0.0001) and mean FC (*p* < 0.0001) and CRP (*p* < 0.0001) levels decreased significantly from baseline during the first 3 months of UPA treatment (Figure 4A). Data on both biomarkers were available for 20/86, 50/84, and 61/97 patients at weeks 2, 8, and 12, respectively. Biochemical remission was reported in 3/20 (15.0%) patients at week 2, in 22/50 (44.0%) patients at week 8, and in 37/61 (55.7%) patients at week 12.

At week 24, 92.5% (62/67) of the patients achieved a clinical response, and 86.6% (58/67) experienced CR. Overall, 80.6% (54/67) of the patients had CSFCR (Figure 3B). Biochemical activity was observed in 20.9% (14/67) of the patients who had CR. Endoscopy was performed in 36 patients, of which 58.3% (21/36) showed an endoscopic response and 36.1% (13/36) had endoscopic remission. Data on clinical, biochemical, and endoscopic outcomes were available in 32/67 patients, and complete remission was achieved by 40.6% (13/32) of these patients.

Data on the therapeutic response were available for all 29 patients at week 52, of which 93.1% (27/29) had CR. CSFCR was documented in 86.2% (25/29) of the patients (Figure 3B). In 22.2% (6/27) of the patients with CR, biochemical activity was detected. Endoscopy was performed in 17 patients. Overall, 64.7% (11/17) of the patients achieved an endoscopic response, while 52.9% (9/17) of the patients showed endoscopic remission. Data on clinical, biochemical, and endoscopic outcomes were available in 17/29 patients, of which 44.4% (8/18) had complete remission.

**Figure 5:**
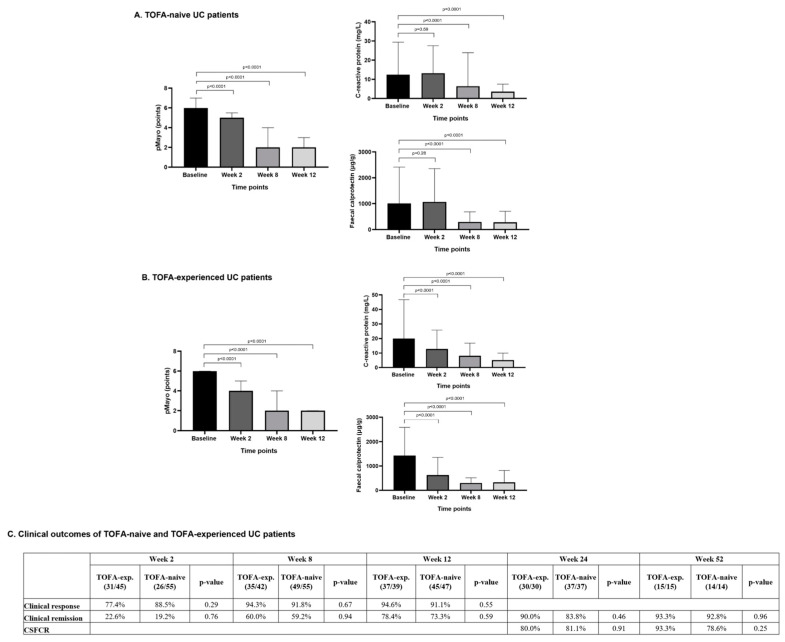
(**A**) A significant reduction in median pMayo and mean CRP and FC was detected over the first 12 weeks of UPA therapy in TOFA-naive UC patients. (**B**) A significant reduction in median pMayo and mean CRP and FC was detected over the first 12 weeks of UPA therapy in TOFA-experienced UC patients. (**C**) The assessment of clinical and biochemical outcomes in TOFA-naive and TOFA-experienced UC patients receiving upadacitinib. There was no statistically significant difference in the clinical endpoints examined (clinical response, clinical remission, CSFCR).

Significant reductions in pMayo and biomarkers were observed in both TOFA-naive and TOFA-experienced patients. No difference in clinical response, CR, or CSFCR rates was found between the two patient groups (Figure 5).

Clinical remission rates stratified by disease extent are presented as Supplementary Material (Table S1).

In 9.4% (3/32) of the UC patients treated with 15 mg UPA, dose escalation was needed, on average, 10.0 (±8.9) months after treatment initiation. IBD-related hospitalisations occurred in 5.0% (5/100) of the patients during the follow-up period, of which three patients underwent colectomy, all for chronically active UC. The probability of treatment persistence with UPA was 92.3% at both weeks 24 and 52. (Figure 3B). Treatment discontinuation was reported in 7.0% (7/100) of the patients, mainly due to PNR (71.4%).

#### 3.3.2. Filgotinib

At week 8, data on the therapeutic response was available for 27/29 patients, of which 74.1% (20/27) achieved a clinical response and 29.6% (8/27) had CR. (Figure 4B). The induction phase was extended to 22 weeks for 14.8% (4/27) of the patients. After the induction phase, 38.5% (10/26) of the patients received a maintenance dose of 100 mg, while 61.5% (16/26) of the patients were on a maintenance dose of 200 mg. At week 2, no data on FC and CRP levels were available for any patients. The mean FC and CRP levels decreased significantly from baseline to week 12 (*p*[FC] = 0.04; *p*[CRP] = 0.04) (Figure 4B). Data on both biomarkers were available for 17/29 and 15/27 patients at weeks 8 and 12, respectively. Biochemical remission was not reported in any patient at week 2 but was observed in 35.3% (6/17) of the patients at week 8 and 40.0% (6/15) of the patients at week 12.

At week 24, 95.5% (21/22) of the patients had a clinical response, and 63.6% (14/22) showed CR. CSFCR was reported in 59.1% (13/22) of the patients. (Figure 3C). In 28.6% (4/14) of the patients with CR, biochemical activity was detected. At week 24, endoscopy was performed in only two patients; both showed an endoscopic response.

Week 52 data on the therapeutic response were available for all patients with sufficient follow-up, of which 80.0% (4/5) achieved CR and 60.0% (3/5) CSFCR (Figure 3C). Biochemical activity was observed in 40.0% (2/5) of the patients who had CR. Four patients had an endoscopy at week 52, of which 60.0% (3/5) had an endoscopic response, while 20.0% (1/5) of the patients achieved endoscopic remission.

Clinical remission rates stratified by disease extent are presented as Supplementary Material (Table S1).

Dose escalation was reported in 20.0% (2/10) of the UC patients treated with 100 mg FILGO, on average 5.9 (±2.3) months after treatment initiation. IBD-related hospitalisation was required for 6.5% (2/31) of the patients during the course of treatment. No surgical intervention was needed during the follow-up period. The probability of treatment persistence with FILGO was 78.5% at week 24 and 61.3% at week 52 (Figure 3C). FILGO was discontinued in 32.3% (10/31) of the patients by the end of the follow-up period, mostly due to LOR (40.0%) and PNR (30.0%).

### 3.4. Safety of Selective JAK Inhibitors

#### 3.4.1. Upadacitinib

A total of 101 AEs (Table 2) were reported in 32.6% (70/215) of the CD and UC patients treated with UPA. AEs were seen in 24.4% (11/45) of the TOFA-experienced UC patients and 47.2% (26/55) of the TOFA-naive UC patients on UPA. The most frequent AEs were hyperlipidaemia (21/215; 9.8%), acne (9/215; 4.2%), and varicella zoster virus (VZV) infection (8/215; 3.7%). One patient (0.5%) was diagnosed with a new-onset melanoma malignum. As a result of AEs, therapy was discontinued in 4.7% (10/215) of the patients, and in 7.4% (16/215), the dose was reduced from 30 mg to 15 mg. The majority of the AEs were reversible (92.1%); however, hyperlipidaemia was present in five patients, and hypertension persisted in three patients during the follow-up period. The univariable logistic regression analysis showed no statistically significant association between the maintenance dosage (15 mg vs. 30 mg) and the occurrence of AEs in the UPA-treated CD (*p* = 0.89) and UC (*p* = 0.43) patients.

#### 3.4.2. Filgotinib

AEs were observed in 6/31 (19.4%) patients receiving FILGO therapy. In total, eight AEs (Table 2) were documented during the follow-up period, of which the most frequent were hyperlipidaemia (3/31; 9.7%) and VZV infection (2/31; 6.5%). Two AEs resulted in treatment discontinuation (6.5%), while dose reduction (from 200 mg to 100 mg) was required in one patient (3.2%). During the follow-up period, hyperlipidaemia persisted in one patient, while the other AEs were reversible (96.8%). No statistically significant association was found between the maintenance dosage (200 mg vs. 100 mg) and the occurrence of AEs in the FILGO-treated UC (*p* = 0.99) patients.

### 3.5. Predictors of Clinical Remission

Various demographic and clinical data, as well as laboratory parameters, were analysed to predict CR at the end of the induction phase. The results are presented as Supplementary Material. Given the low number of patients receiving FILGO treatment, no predictive analysis was performed.

#### 3.5.1. Crohn’s Disease

The CD patients who had a complicated disease behaviour (stricturing and/or penetrating; OR:0.28; 95% CI: 0.08,0.97; *p* = 0.04) and/or higher CDAI at treatment initiation (OR:0.94; 95% CI: 0.89,0.99; *p* = 0.02) were less likely to achieve CR with UPA by week 12. (Table S2).

#### 3.5.2. Ulcerative Colitis

The UC patients with younger ages (OR:1.00; 95% CI: 1.00,1.01; *p* = 0.03) and/or higher pMayo (OR:0.42; 95% CI: 0.21,0.83; *p* = 0.01) at treatment initiation were less likely to achieve CR with UPA by week 8. Among other factors, prior TOFA exposure (*p* = 0.91) did not affect the outcome (Table S3).

## 4. Discussion

In this retrospective, multicentre observational study, we provide real-world data demonstrating the effectiveness and safety of selective JAKis in moderate-to-severe CD and UC patients. Based on our results, selective JAKis are highly effective both in refractory CD and UC patients. Although a beneficial effect on the clinical and biochemical disease activity was detected as early as 2 to 8 weeks post-initiation, the peak effectiveness of selective JAKis was reached between 3 and 6 months. We found that CD patients who had complicated disease behaviour and/or higher CDAI at treatment initiation were less likely to achieve CR with UPA by week 12. UPA-treated UC patients with younger ages and/or higher pMayo were also less likely to achieve CR by week 8. Importantly, failure to prior TOFA did not have any effect on the therapeutic outcome of UPA in UC.

There is a dearth of real-world studies investigating the effectiveness and safety of selective JAKis, with most of our data on the efficacy of selective JAKis coming from phase II/III randomised controlled trials [16,17,18,19,20]. The optimal, rigorously controlled environment in clinical trials limits the real-world generalisability of therapeutic outcomes of UPA and FILGO. Investigating selective JAKis in real-world settings is crucial to gaining insight into the effectiveness and safety of these agents in a more diverse patient population and to adequately position them in the therapeutic algorithm.

Limitations posed by clinical trials notwithstanding, the lack of consistency with the definitions used for CR make direct comparison of the available data challenging. Two previously published retrospective studies, involving 93 and 45 CD patients, reported CR rates of 27.3% and 64.0% at week 12. In the first cohort, CR rates were 48.0% and 38.0% at weeks 24 and 52, respectively [21,22]. The remission rates in our refractory cohort were substantially higher: 76.2%, 80.2%, and 79.2% at weeks 12, 24, and 52, respectively. In a prospective study of 45 bio-experienced CD patients, 56.2% and 70.6% of the patients were in CR at weeks 2 and 8, as compared to 22.5% and 29.3% in our cohort [23]. To date, only one real-world study has been published on the effectiveness of UPA in UC [23]. In a prospective cohort of 44 refractory UC patients, CR was achieved in 36.0% and 85.2% of the patients at weeks 2 and 8. CR was observed in 21.1% and 59.5% of the patients at the same endpoints. In a retrospective study [24] of 91 UC patients receiving FILGO, the rates of CR were 71.9% and 76.4% at weeks 12 and 24, compared to the CR rates of 57.1% and 63.6% observed at weeks 12 and 24 in the present study involving only refractory patients. Based on the results of another retrospective cohort involving 238 UC patients, the CR rate was 47%, 55.8%, and 64.6% at weeks 10, 26, and 58, respectively [25]. Overall, 53.2% of the patients achieved CSFCR at week 26 and 60.9% of the patients at week 58. Although our results are not directly comparable due to the differences in the time points studied, the higher CR rates obtained in our study are likely due to the different definitions used.

According to the treat-to-target approach, the effectiveness of the agent should be characterised by more stringent endpoints, such as endoscopic remission and the normalisation of biomarkers, apart from achieving CR [33]. Despite the encouragingly high CR rates demonstrated in the present study, around one-third of the FILGO-treated UC patients in CR showed biochemical activity at weeks 24 and 52. Nearly half of the asymptomatic CD patients showed biochemical activity with UPA treatment, while one-fifth of the UC patients in CR had elevated FC and/or CRP. In addition to demonstrating the effectiveness of selective JAKis in reducing IBD symptoms, these findings also highlight the importance of tight biomarker monitoring to achieve long-term success. The endoscopic response was only ever evaluated in one real-world study. Endoscopic remission at weeks 24 and 52 was detected in 28.3% and 54.5% of the UPA-treated CD patients and in 36.1% and 52.9% of the UC patients receiving UPA. Consistent with our results, Chugh et al. found that 28.6% of CD patients treated with UPA achieved endoscopic remission at week 24 [21]. Based on our data, the rate of complete remission with UPA was around 40% in both the CD and UC patients at the same time points. In the present study, 0.0% and 20.0% of the FILGO-treated UC patients achieved endoscopic remission at the same time points; however, these results should be interpreted with caution due to the small number of patients undergoing colonoscopy.

Assessing the changes in biochemical markers after treatment initiation may provide an objective measure of effectiveness. In line with our findings, the majority of previously published studies detected a significant reduction in biomarkers, such as CRP and FC, within 3 months of selective JAKi therapy [22,23,24,25]. In contrast, Chugh et al. found no statistically significant difference in FC during the first 3 months of UPA therapy in CD patients (*p* = 0.18) [21]. The rate of biochemical remission with the use of selective JAKis has previously only been investigated in one real-world cohort (defined as CRP ≤ 5 mg/L and FC < 250 µg/g) [24], in which it was observed in 87.3% of FILGO-treated UC patients, as compared with 40.0% or 60.0% in our study, depending on which definition is used (CRP ≤ 5 mg/L FC ≤ 150 or ≤250 µg/g).

There is a dearth of data on treatment persistence with selective JAKis. In a UK cohort of CD patients [22], the probability of treatment persistence with UPA was 87.1% at week 24 and 81.7% at week 52, which is almost identical to the 90.5% and 79.0% persistence rates detected in the present study at the same endpoints. Gros et al. found that 82.4% of patients remained on FILGO at the median follow-up of 42 weeks [24]. In the present cohort, including only refractory UC patients, the probability of persistence with FILGO was slightly lower: 78.5% and 61.3% at weeks 24 and 52, respectively.

In clinical practice, physicians may encounter UC patients who have not achieved or maintained the desired outcome with any of the currently available biologics or TOFA. Patients previously exposed to TOFA were not included in the U-ACHIEVE trials, underpinning the need for further studies to evaluate the effectiveness of selective JAK1 inhibitors in UC patients who have failed prior TOFA therapy [18,34]. Both the data reported by Friedberg et al. and the results of the present study suggest that the use of UPA may be beneficial even after the failure of panJAKi [23].

Notably, our results suggest that UPA-treated CD patients with complicated disease behaviour are less likely to achieve CR by week 12. Given the lack of data on the effectiveness of UPA in CD patients with a complicated disease phenotype, further studies with these specific patient groups are needed.

The use of selective JAKi may be supported by the hypothesis that they may have a better safety profile than TOFA due to their specific affinity for JAK1. Based on the data from a recent systematic review and meta-analysis of randomised controlled trials and real-world observational studies, the pooled rate of AEs in UC patients was 65% and 75% in CD patients receiving UPA [35]. Considering only real-world studies, the incidence of AEs ranges from 27% to 40% [21,22,23]. In the SELECTION trial, the incidence of FILGO-related AEs was 60.3% and 66.8% in patients receiving a maintenance dose of 100 and 200 mg, which is substantially higher than detected in real-world studies (9.2–16.5%) [16,24,25]. Meanwhile, our real-world data showed that the incidence of AEs was 47.0% in the UPA-treated CD and UC cohorts, whereas it was 25.8% in the FILGO-treated UC cohort. In both patient groups, the most common AEs were hyperlipidaemia and VZV infection. No major adverse cardiovascular events or deaths were reported with selected JAKi during the follow-up period; however, one patient was diagnosed with melanoma skin cancer (0.5%) following the initiation of UPA. Previous studies have also shown a significant increase in total and low-density cholesterol levels with the use of JAKis [19,36,37]. While all JAKis increase the risk of VZV infection through the inhibition of JAK1 and thus IFN suppression [11], a systematic review and network meta-analysis showed that IBD patients who receive JAKis are more likely to acquire the infection than those being treated with other biologics [38]. The high rate of VZV infection with selective JAKis observed in our cohort may be explained by the fact that only a small proportion of the IBD patients were clearly vaccinated against VZV.

We acknowledge some limitations in our study. The key limitation is its retrospective nature, which introduces confounding through inherent variability in assessments. The retrospective study design precluded the random allocation of treatment groups, and with a limited number of previous studies, sample size calculation was not possible. Although the participating centres included all the patients eligible for enrolment in this study, selection bias may have been introduced by the strict inclusion criteria and by differences in patient numbers across the centres. The effect of selective JAKis on perianal fistulas was not assessed by radiological endpoints, and the potential impact on EIMs was not investigated. Histological endpoints were not studied. Data on clinical, biochemical, and endoscopic outcomes were missing in some patients due to the retrospective study layout. Therefore, the outcomes were only assessed in patients with available data at the predetermined time point, which may have resulted in generally higher response rates.

Our study has several notable strengths. To the authors’ knowledge, this is the largest real-world multicentre study to date that has investigated and demonstrated the effectiveness and safety of selective JAKis in refractory UC and CD patients with moderate-to-severe disease activity. In the present study, we also evaluated biochemical and endoscopic outcomes that were generally lacking in previous studies. The potential impact of switching from TOFA to UPA on effectiveness was also assessed. Multivariable logistic regression analyses were applied to determine independent predictors of CR at the end of the induction phase.

## 5. Conclusions

Selective JAK inhibitors are effective and safe for treating refractory, moderate-to-severe UC and CD patients. Significant reduction in clinical and biochemical disease activity was observed in the majority of patients by the second week of treatment. In UC, the effectiveness of UPA appears not to be compromised by prior TOFA failure, while UPA-treated CD patients with complicated disease behaviour are less likely to achieve CR by the end of the induction phase. The three most common AEs were hyperlipidaemia, acne, and VZV infection, all with a frequency of less than 10%.

Despite the encouraging data observed in the present study regarding the effectiveness of selective JAK inhibitors in IBD patients, prospective controlled trials are needed to confirm our findings and to adequately position these agents in the therapeutic algorithm.

## Figures and Tables

**Table 1:** Baseline demographic and clinical data of the cohort study.

Demographic and Clinical Characteristics (*n* = 246)				
	UC (*n* = 131)			CD (*n* = 115)
	UPA (*n* = 100)		FILGO (*n* = 31)	
	TOFA-Experienced (n = 45)	TOFA-Naive (n = 55)		
Male (*n*;%)	29 (64.5%)	35 (63.6%)	12 (38.7%)	57 (49.6%)
Age at the diagnosis (years; mean ± SD)	28.5 ± 12.1	30.7 ± 12.7	34.6 ± 14.7	27.1 ± 14.6
Follow-up time (months; median [IQR]; mean ± SD)	8.7 ± 4.7; 7.7 [11.7–5.4]			8.3 ± 4.4; 7.3 [11.2–5.1]
The number of prior therapeutic lines (median [IQR])	3 [3–2]	2 [3–1]	2 [2–1]	3.0 ± 1.1
Prior anti-TNF failure (*n*;%)	96 (96.0%)		26 (83.9%)	109 (94.8%)
Prior failure of at least two agents with different mechanisms of action (*n*;%)	77 (77.0%)		16 (51.6%)	96 (83.5%)
Bowel resection prior to selective JAKi (*n*;%)				27 (23.5%)
Age at selective JAKi initiation (years; mean ± SD)	38.4 ± 12.7	40.6 ± 14.9	43.5 ± 15.3	40.0 ± 14.8
Disease duration at selective JAKi initiation (years; mean ± SD)	10.0 ± 6.8	9.9 ± 7.7	9.0 ± 9.7	13.4 ± 9.7
Serum albumin level at selective JAKi initiation (mean ± SD)	37.5 ± 5.7	37.7 ± 5.7	37.6 ± 5.3	40.7 ± 42.2
BMI at selective JAKi initiation (mean ± SD)	22.6 ± 3.2	24.1 ± 4.5	23.6 ± 4.8	23.1 ± 5.0
Extraintestinal manifestations at selective JAKi initiation (*n*;%)				
Hepatobiliary disease	2 (4.4%)	2 (3.6%)	0 (0.0%)	5 (4.4%)
Musculosceletal	4 (8.9%)	12 (21.8%)	5 (16.1%)	38 (33.0%)
Ocular	0 (0.0%)	0 (0.0%)	1 (3.2%)	2 (1.7%)
Dermatologic	1 (2.2%)	5 (9.1%)	1 (3.2%)	8 (7.0%)
Extent of the disease at selective JAKi initiation, if available—(*n*;%) †				
Proctitis	1 (2.2%)	4 (7.4%)	3 (9.6%)	
Distal colitis	13 (28.9%)	17 (30.9%)	14 (45.2%)	
Pancolitis	25 (68.9%)	34 (61.8%)	14 (45.2%)	
Ileal				15 (13.0%)
Colonic				30 (26.1%)
Ileocolonic				70 (60.9%)
Isolated upper disease				0 (0.0) ‡
Perianal involvement (*n*;%)				19 (16.5%)
Disease behaviour at selective JAKi initiation (*n*;%) †				
Inflammatory				74 (64.3%)
Stricturing				33 (28.7%)
Penetrating				8 (6.6%)
Endoscopic disease activity at selective JAKi initiation, if available—(*n*;%)				
eMayo 1	0 (0.0%)	0 (0.0%)	4 (14.8%)	
eMayo 2	15 (37.5%)	19 (38.0%)	16 (59.3%)	
eMayo 3	25 (62.5%)	31 (62.0%)	7 (25.9%)	
SES-CD 0–2				0 (0%)
SES-CD 3–6				0 (0%)
SES-CD 7–15				70 (93.3%)
SES-CD ≥ 16				5 (6.7%)
Concomitant corticosteroid use at selective JAKi initiation (*n*;%)	17 (37.8%)	11 (20.0%)	6 (19.4%)	33 (28.7%)

† Montreal classification. ‡ A total of 13 patients had upper gastrointestinal involvement.

**Table 2:** Adverse events during the use of selective JAK inhibitors.

Upadacitinib (*n* = 215)	
Patients affected (*n*;%)	70 (32.6%)
Adverse events (*n*;%)	101 (46.8%)
Nasopharyngitis (*n*;%)	5 (2.3%)
Viral pneumonia (*n*;%)	1 (0.5%)
Urinary tract infection (*n*;%)	2 (0.9%)
VZV infection (*n*;%)	8 (3.7%)
HSV infection (*n*;%)	4 (1.9%)
Fever without verified infection (*n*;%)	7 (3.3%)
Leukopenia without verified infection (*n*;%)	5 (2.3%)
Neutropenia without verified infection (*n*;%)	4 (1.9%)
Acne (*n*;%)	9 (4.2%)
Hyperlipidaemia (*n*;%)	21 (9.8%)
Hyperuricaemia (*n*;%)	2 (0.9%)
Hyperglycaemia (*n*;%)	1 (0.5%)
Elevated serum creatinine levels (*n*;%)	
<10% elevation	1 (0.5%)
10–20% elevation	2 (0.9%)
Nausea (*n*;%)	4 (1.9%)
Vomiting (*n*;%)	1 (0.5%)
Abdominal pain (*n*;%)	5 (2.3%)
Rectal bleeding (*n*;%)	3 (1.4%)
Myalgia (*n*;%)	2 (0.9%)
Arthralgia (*n*;%)	7 (3.3%)
Hypertension (*n*;%)	7 (3.3%)
Filgotinib (*n* = 31)	
Patients affected (*n*;%)	6 (19.5%)
Adverse events (*n*;%)	8 (25.8%)
Nasopharyngitis (*n*;%)	1 (3.2%)
Urinary tract infection (*n*;%)	1 (3.2%)
VZV infection (*n*;%)	2 (6.5%)
Hyperlipidaemia (*n*;%)	3 (9.7%)
Angina (*n*;%)	1 (3.2%)

## Data Availability

The raw data that support the findings of this study are not publicly available, in accordance with the EU General Data Protection Regulation’s (GDPR) guidelines for processing personal data. Data are only available upon reasonable request from the corresponding author.

## Supplementary Materials

The following supporting information can be downloaded at https://www.mdpi.com/article/10.3390/jcm13247804/s1: Table S1: Clinical remission rates stratified by disease location/extent; Table S2: The results of the uni- and multivariable logistic regression models for the prediction of clinical remission at week 12 in patients with Crohn’s disease, receiving upadacitinib; Table S3: The details of the univariable logistic regression models for the prediction of clinical remission at week 8 in patients with ulcerative colitis receiving upadacitinib.
