# Supplementary material for: Real-World Effectiveness and Safety of Selective JAK Inhibitors in Ulcerative Colitis and Crohn’s Disease: A Retrospective, Multicentre Study
Source: J Clin Med. 2024 Dec 20;13(24):7804. doi: 10.3390/jcm13247804 (PMC11728011; doi:10.3390/jcm13247804)
Supplement: Supplementary file 1 [file jcm-13-07804-s001.zip › jcm-3323227-supplementary.pdf]

Supplementary Material

Table S1. Clinical remission rates stratified by disease location/extent.

**Upadacitinib**	**Clinical remission rates**				
**Disease location - Crohn's disease**	**Week 2**	**Week 8**	**Week 12**	**Week 24**	**Week 52**
Ileal †	8.3% (1/12)	15.4% (2/13)	78.6% (11/14)	76.9% (10/13)	-
Colonic	16.7% (3/18)	45.8% (11/24)	77.8% (21/27)	83.3% (20/24)	85.7% (6/7)
Ileocolonic ‡	16.0% (8/50)	25.5% (14/55)	71.4% (40/56)	79.6% (43/54)	76.5% (13/17)

**Upadacitinib**	**Clinical remission rates**				
**Disease extent - Ulcerative colitis**	**Week 2**	**Week 8**	**Week 12**	**Week 24**	**Week 52**
Pancolitis	18.4% (7/38)	57.1% (32/56)	71.9% (41/56)	84.8% (39/46)	95.0% (19/20)
Left sided colitis	21.4% (3/14)	62.5% (15/24)	76.2% (16/21)	88.9% (16/18)	100.0% (7/7)
Proctitis	40.0% (2/5)	50.0% (2/4)	100.0% (5/5)	100.0% (3/3)	50.0% (1/2)

**Filgotinib**	**Clinical remission rates**				
**Disease extent - Ulcerative colitis**	**Week 2**	**Week 8**	**Week 12**	**Week 24**	**Week 52**
Pancolitis	12.5% (1/8)	30.8% (4/13)	61.5% (8/13)	54.6% (6/11)	66.7% (2/3)
Left sided colitis	0.0% (0/4)	33.3% (4/12)	30.8% (4/13)	63.6% (7/10)	100.0% (2/2)
Proctitis	0.0% (0/2)	0.0% (0/2)	0.0% (0/2)	100.0% (1/1)	-

† 2 patients had upper gastrointestinal involvement

‡ 11 patients had upper gastrointestinal involvement

Table S2. The results of the uni- and multivariable logistic regression models for the prediction of clinical remission at week 12 in patients with Crohn's disease, receiving upadacitinib. Hosmer and Lemeshow Test $X^2 = 3.69$, $p = 0.88$, Nagelkerke $R^2 = 0.15$

Week 12 clinical remission - CD	Univariable				Multivariable			
	p-value	OR	95% CI		p-value	OR	95% CI	
Sex	0.72	1.18	0.48	2.88				
No smoking	0.35	2.14	0.43	10.6				
Active smoker	0.99	>10.0	0.00					
Former smoker	0.96	0.96	0.27	3.48				
The number of prior biological therapies	0.60							
One vs Two-lines of biologics	0.44	0.40	0.04	4.10				
One vs Three-lines of biologics	0.47	0.43	0.04	4.35				
One vs. Four-lines of biologics	0.80	0.15	0.18	1.28				
Age at treatment initiation	0.94	0.99	0.96	1.04				
Disease duration	0.12	0.96	0.90	1.01	0.43	0.97	0.91	1.04
CD location at treatment initiation	0.65							
Ileal vs. Colonic	0.40	1.94	0.47	8.05				
Ileal vs. Ileocolonic	0.60	1.39	0.40	4.79				
Small bowel involvement	0.45	0.67	0.24	1.89				
**Complicated disease behavior (stricturing and penetrating CD) at treatment initiation**	0.11	0.47	0.19	1.17	**0.04**	**0.28**	**0.08**	**0.97**
Perianal involvement	0.26	0.52	0.16	1.62				
**CDAI at treatment initiation**	0.09	0.96	0.92	1.00	**0.02**	**0.94**	**0.89**	**0.99**
Serum albumin at treatment initiation	0.48	0.97	0.89	1.06				
CRP at treatment initiation	0.86	0.99	0.99	1.01				
Fecal calprotectin at treatment initiation	0.28	1.00	1.00	1.00				
SES-CD at treatment initiation	0.37	1.10	0.90	1.34				
Concomitant corticosteroids at treatment initiation	0.66	0.79	0.28	2.24				

Abbreviations: CD= Crohn's disease; CDAI= Crohn's disease activity index; CI: confidence interval; CRP= C-reactive protein; OR= odds ratio; SES-CD= Simple endoscopic score for Crohn's disease, vs.= versus

Table S3. The details of the univariable logistic regression models for the prediction of clinical remission at week 8 in patients with ulcerative colitis, receiving upadacitinib. Hosmer and Lemeshow Test $X^2 = 3.47$, $p = 0.84$, Nagelkerke $R^2 = 0.30$

Week 8 clinical remission - UPA-treated UC	Univariable				Multivariable			
	p-value	OR	95% CI		p-value	OR	95% CI	
Sex	0.51	0.73	0.29	1.86				
No smoking	0.27	0.51	0.16	1.68				
Active smoker	0.98	1.03	0.09	12.1				
Former smoker	0.34	0.51	0.13	1.99				
The number of prior biological therapies	0.28							
One vs Two-lines of biologics	0.20	0.45	0.13	1.54				
One vs Three-lines of biologics	0.20	0.48	0.16	1.47				
One vs. Four-lines of biologics	0.62	1.48	0.32	6.88				
Prior TOFA administration	0.91	0.95	0.39	2.31				
**Age at treatment initiation**	0.10	1.00	1.00	1.01	**0.03**	**1.00**	**1.00**	**1.01**
Disease duration	0.73	1.01	0.95	1.08				
Disease extent at treatment initiation	0.58							
Proctitis vs left sided colitis	0.99	0.00	0.00					
Proctitis vs pancolitis	0.99	0.00	0.00					
Serum albumin at treatment initiation	0.54	1.02	0.94	1.14				
CRP at treatment initiation	0.49	0.99	0.97	1.01				
Fecal calprotectin at treatment initiation	0.10	1.00	1.00	1.01	0.46	1.00	1.00	1.00
**pMayo at treatment initiation**	0.002	0.45	0.26	0.75	**0.01**	**0.42**	**0.21**	**0.83**
eMayo at treatment initiation	0.29	0.62	0.26	1.50				
Concomitant corticosteroids at treatment initiation	0.26	1.79	0.66	4.86				

Abbreviations: CI: confidence interval; CRP= C-reactive protein; eMayo= endoscopic Mayo subscore; OR= odds ratio; pMayo= partial Mayo score; TOFA= tofacitinib; UPA= upadacitinib; vs.= versus
